# Cortical Spreading Depression Elicited in Rat Brain after Exposure to Microwave from GSM Mobile Phone

**Published:** 2006-06

**Authors:** Samera M. Sallam

**Affiliations:** *Department of Physics, Faculty of Science, Benha University, Benha, Egypt*

**Keywords:** cortical spreading depression, global system for mobile communication, slow potential change

## Abstract

The aim of the present work is to evaluate possibility of microwave emitted by cellular phone that can elicit cortical spreading depression (CSD) in rat brain and studying the characteristics of the evoked signals. (CSD) was elicited in cerebral cortex of anesthetized rats after exposure to microwave irradiation (935.2-960.2 MHz) from Global System for Mobile communications (GSM) mobile phone. With the microwave output of about 8.5 mW at the antenna - tissue surface (4mm in diameter), CSD was elicited after 50 sec irradiation from the beginning of a received signal to the mobile and after 35 sec irradiation from the beginning of a transmitted signal from the mobile. CSD was elicited in about 90% of experiments after irradiation by both types of signal exposure. The results have shown that slow potential change (SPC) has an amplitude of 4.5 ± 0.75 mV, duration of 1.5 ± 0.5 min and propagated speed of 3 mm/min on the average. The amplitude, duration and behaviour of SPC of the evoked spreading depression were found to be affected by irradiation time and the method of exposure.

## INTRODUCTION

In higher mammals, the cerebral cortex is the highest and most important part in the central nervous system. Spreading depression (SD) is employed as a tool in behavioral studies and in research into the functional organization of brain. Such phenomenon becomes important not only for research but also for diagnostic and therapeutic purposes. It may underlie certain clinical neurological and pathological conditions, propagates in the manner of a wave invades the tissue surface (1-3). It has been suggested that its appearance is associated with seizures, ischemia, head injury (4). It can be experimentally triggered by high frequency electrical pulses or direct current, mechanical stimuli such as pressure on the cortex, alkaline pH, low osmolarity and variety of chemicals. It has been found that ultrasound can elicit SD (5, 6).

The effect of microwave and electromagnetic fields on brain activity, structural and behavioral changes are thoroughly studied (7-10). But using microwave as an agent for eliciting or antagonizing SD and studying its effects on characteristics of slow potential change (SPC) are deficient in literature. So far, no comprehensive study had evaluated the SD and its accompanied changes under the thermic and nonthermic effects of microwave sources, especially during mobile phone usage. Possibility that microwave energy absorbed by brain tissue may be converted into heat, this must be taken in consideration. Microwave, from global system for mobile communication (GSM) mobile phone, may be used for producing restricted brain damage, but low intensities may produce reversible effects such as improving pathological condition (11). The aim of present work is to study the possibility that microwave emitted be cellular phone (935.2-960.2 MHz) can elicit CSD in rat brain and to study characteristics of the evoke signals.

## METHODS

**Figure 1:**
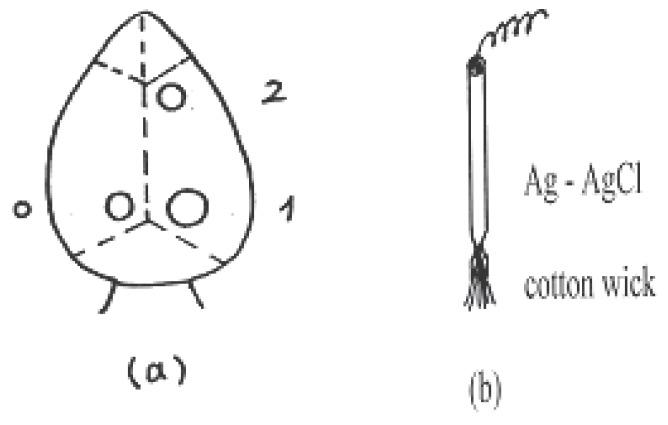
a) The trephine opening over the cerebral cortex; 0-for reference electrode, 1-for stimulation, and 2-for recording SPC; b) The construction of wick Ag-AgCl electrode.

**Figure 2:**
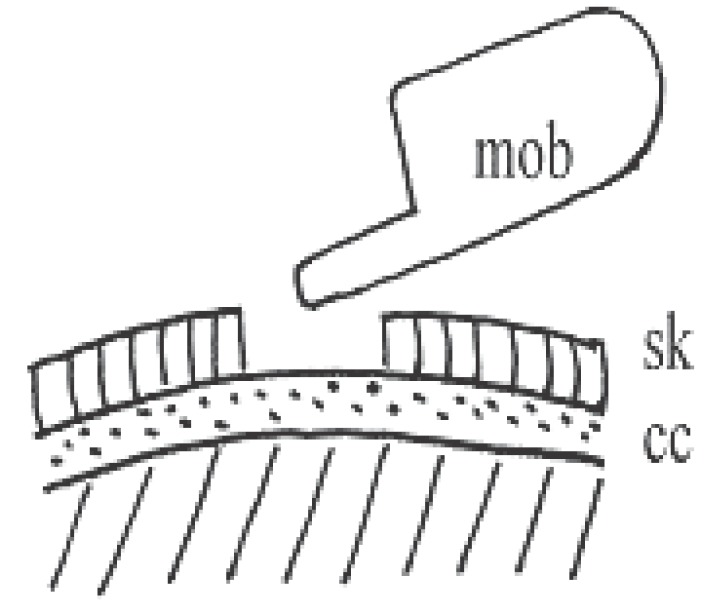
Scheme of the trephine opening through the skull (sk) over the cortical surface (cc) and the mobile antenna (mob).

The experiments were carried out with a total of 60 adult albino rats weighting 100 g on average that were purchased from the holding company for biological products and vaccine, Cairo, Egypt. The rats used in the present work complied with legal requirements and institutional guidelines. The rats were housed individually in plastic boxes kept in shielded chamber to reduce the normal ambient environmental electric field under similar conditions of temperature, illumination, acoustic noise and ventilation and received the same diet during the course of experiments as in Fadel *et al* (12). The rats were anesthetized by subcutaneous injection with pentobarbital 40 mg/kg and the vital condition of the anesthetized rats was monitored by its normal breathing. Under anesthesia, the skin of the head was cut and cerebral cortex was exposed by two trephine openings over the parietal (3 mm in diameter) and occipital (5 mm in diameter) regions of the right hemisphere. These openings were made for stimulation (occipital) and recording SPC (parietal) as shown in Fig. 1a. Trephine opening (3 mm in diameter) was made over the left hemisphere for the reference electrode. Wick Ag-AgCl electrodes (Fig. 1b) were used which are rested gently on the cortical surface avoiding any mechanical stress or damage to the cortical surface. Ringer saline solution at room temperature of 22°C was used for washing the cortical surface from time to time to protect it from drying. The stimulation process was carried out by: (a) application of 2% KCl solution using a piece of filter paper (2 mm in diameter) soaked with the solution for eliciting SD as control experiments, or (b) microwave irradiation signals (935.2-960.2 MHz) received to or emitted from mobile phone which kept silent without ringing during irradiation. The microwave irradiation was carried out by directing the mobile antennae 1cm apart over the occipital opening (Fig. 2).

The SPC accompanying the SD wave was recorded from anesthetized normothermic rats with an electrode placed in the parietal opening (marked 2 in Fig. 1a), relative to a common reference electrode placed in the opening of the left hemisphere (marked 0 in Fig. 1a). In experiments of recording SPC the two electrodes were connected through an interface (PASCO 6500) with a computer program, while in experiments for measuring the propagation velocity of SD an ink-writer (Model CQ 75 saffron waldin – England) was used for better resolution. The SD propagation speed was calculated by knowing the distance between the occipital and parietal openings and the time of SD wave propagation which appear from the recorded SD pattern. Each experiment was repeated six times under the same conditions.

In the present study, similar method as that exploited by Ueda *et al*. (5) was adopted for calculating the intensity of microwave stimulus and the energy output. These parameters were calculated in comparison with similar temperature change aroused from microwave irradiation in contact with 1 ml volume of distilled water isolated in thin-walled plastic vessel.

The statistical method and analysis of the results have been done by calculating the arithmetic mean and standard deviation for all obtained measurements.

## RESULTS

**Figure 3:**
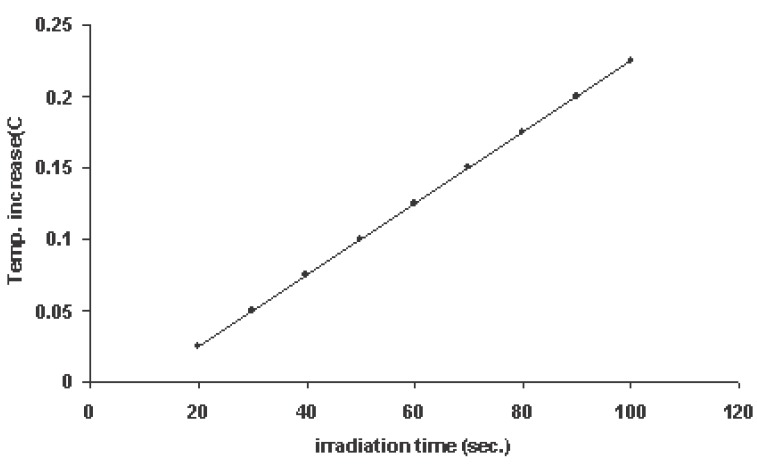
Calibration of microwave stimulus; irradiation time against temperature increase of 1ml water. Each point represents a mean of five records and the standard deviations where found to vary between 6-11%.

Intensity of microwave stimulus: In present work, an experiment was carried out to calibrate the microwave intensity stimulus. 1 ml of distilled water in thin walled polyethylene vessel of cross-sectional area 0.2 cm^2^ was exposed to the microwave emitted from the mobile phone. The increase of temperature of water was measured for different irradiation time, using copper-constantan thermocouple connected to digital thermometer (Roline R0-1310 type-k). Each experiment was repeated five times for each irradiation time and the mean value and the standard deviation were estimated. The results of measurement are shown in Figure 3 together with the computed regression line. After 50 sec irradiation of 1 ml water, its temperature increased by 0.1°C and this corresponds to 0.1 cal or 0.418 Joule. The total microwave output power from the mobile antenna was 8.36 mW and taking into account the area of water contact the power density was 41.8 mW/cm^2^. The results of measurements, for temperature increase with irradiation time is plotted in Fig. 3.

**Figure 4:**
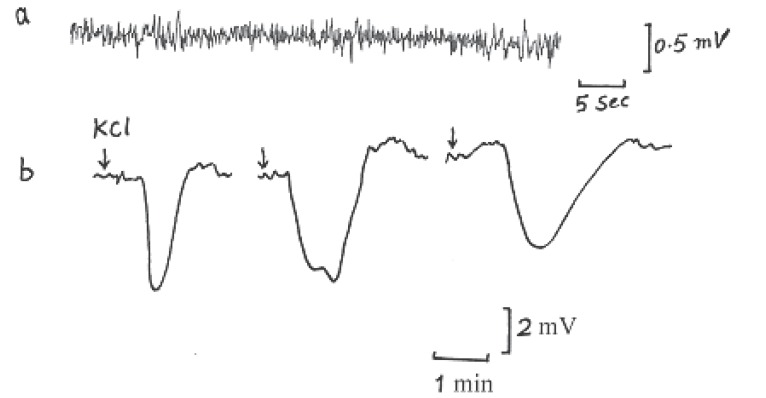
Typical records of (a) cortical activity and (b) CSD wave elicited by 2% KCl in anesthetized normothermic rats (the arrows indicate the site of stimulations).

**CSD elicited by 2% KCl:** Set of experiments has been carried out on 5 anesthetized rats to elicit CSD by 2% KCl. In 25 trials CSD was elicited and some typical records of cortical activity signals and SD waves are shown in Fig. 4. The analysis and measurements of SPCs showed that the mean values of amplitude, duration and propagated speed were 4.5 ± 0.75 mV, 1.5 ± 0.5 min and of 3 ± 0.2 mm/min respectively.

**CSD elicited by microwave irradiation:** 40 rats were used for studying the characteristics, percent of appearance and reproducibility of elicited CSD due to irradiation by microwave signals received by and transmitted from the mobile phone (20 rats for each type of irradiation).

**Figure 5:**
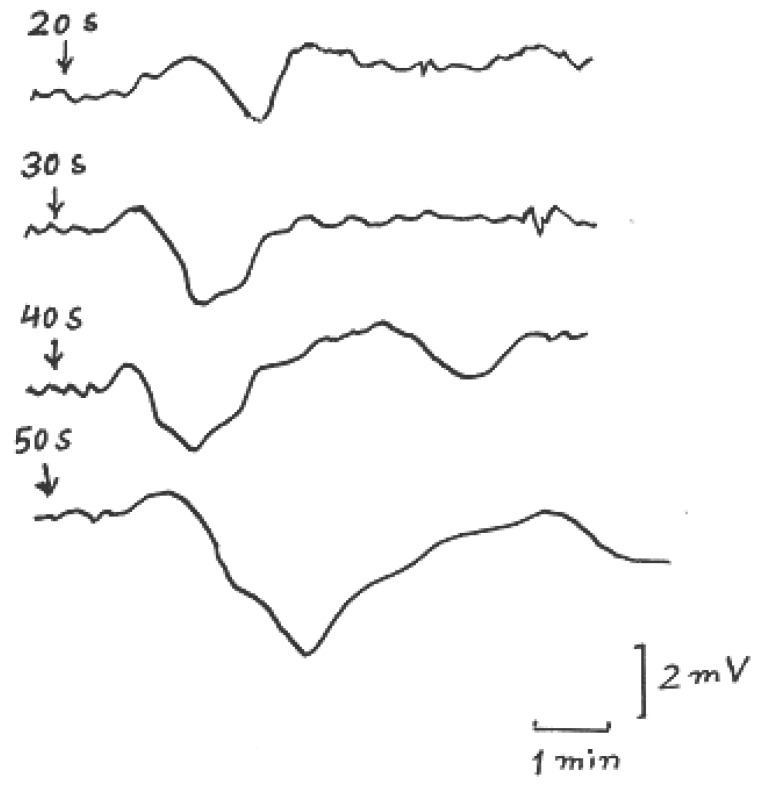
Typical records of CSD for different microwave irradiation periods 20, 30, 40, and 50 Sec. due to exposure of signals received by the mobile (the arrows indicate the site of exposure).

Figure 5 shows typical records of CSD induced in exposed cerebral cortex of anesthetized rats due to microwave irradiation received by the mobile phone for different exposure time. In 25 experiments of this type of irradiation no SD occurred due to exposure time lasting less than 20 sec. So after by increasing irradiation time in 10 sec increments at 10 min intervals the percent of appearance of the SD was increased but with SPCs have small amplitude and duration. At microwave stimulation lasting 50 sec, SD appeared in 90% of experiments with SPC amplitude 4 ± 0.75 mV and duration 2.5 ± 0.5 min and propagation speed of 3 mm/min on the average. By increasing the irradiation time the duration of SPC increased while its amplitude and propagation speed remained approximately constant. The irradiation time of 50 sec used as a threshold value for calculation of calorimetrically estimated energy output of microwave stimulus with the guidance of calibration curve of Figure 3.

**Figure 6:**
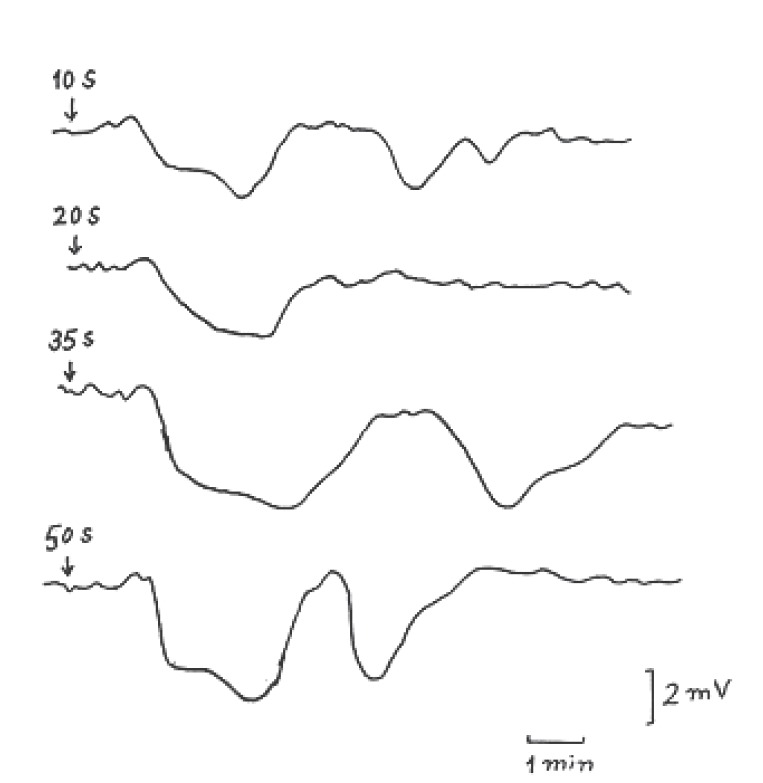
Typical records of CSD evoked by microwave signals emitted from the mobile for exposure periods of 10, 20, 35 and 50 sec. as indicated by the arrows.

Figure 6 illustrates typical CSD records elicited in exposed cerebral cortex of anesthetized rats due to microwave irradiation signals transmitted from the mobile phone. In 25 experiments of this type of irradiation on 20 rats, SD was evoked in 40% of trials due to the effect of stimulus lasting 10 sec accompanied by SPC having low amplitude and longer duration. By increasing the stimulus duration, the percentage of SD appearance increased and reached about 90% at irradiation time of 35 sec showing repetitive SPC characterized by an amplitude of 3.25 ± 0.15 mV and long duration of 3.75 ± 0.25 min with convulsive shapes in most records.

**Figure 7:**
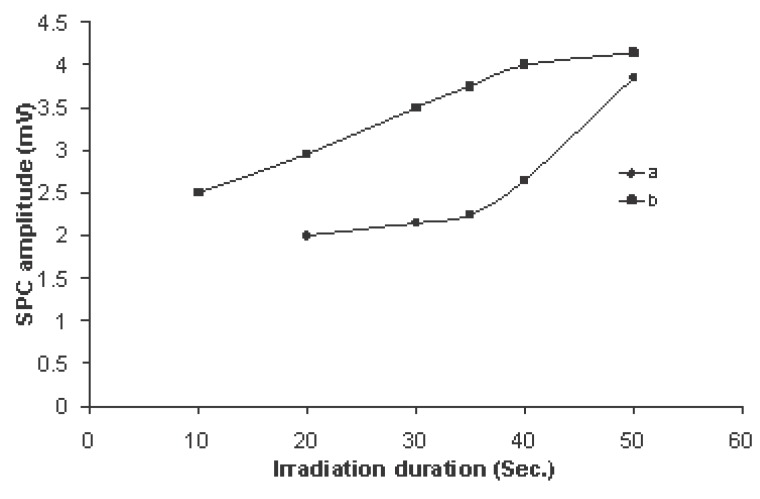
Variation of SPC amplitude with irradiation times of exposure 10, 20, 30, 40 and 50 sec for (a) received signal to the mobile (b) transmitted signal from the mobile. (Each point on the curves represents a mean of five records and the standard deviation found to vary between 8-15%).

In all previous measurements on 40 rats, for both types of irradiation, the amplitude and duration of SPCs were measured from the recorded patterns and the average reading of five runs were used to calculate the mean and the standard deviation for each irradiation time. Figure 7 shows the variation of SPC amplitude with irradiation times in both types of irradiation. The amplitude of SPC showed an increase with increasing the irradiation time, but the amplitude values in case of transmitted signals were about 50% greater than those in case of received signals for irradiation time up to 40 sec. By increasing the irradiation time greater than 40 sec the amplitude of SPC evoked by the received signal approached that of the transmitted signal having values of 3.75 ± 0.25 mV and 4.25 ± 0.65 mV respectively at 50 sec of irradiation.

**Figure 8:**
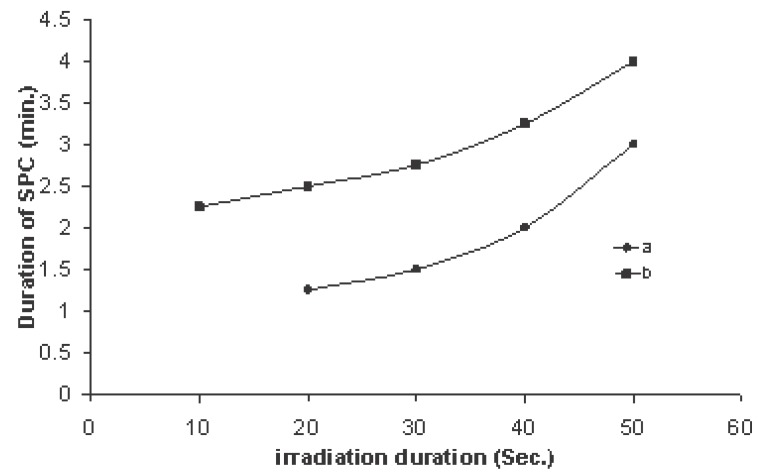
Variation of SPC duration time with irradiation times of exposure 10, 20, 30, 40 and 50 sec for (a) received signal to the mobile (b) transmitted signal from the mobile. (Each point on the curves represents a mean of five records and the standard deviation found to vary between 6-13%).

Figure 8 shows the variation of SPC duration with microwave irradiation time for both types of irradiation. The results showed an increase in duration with the irradiation time, but the duration of SPC evoked by transmitted signal exposure was about 75% greater than that evoked by received signal exposure for the same irradiation time having the values of 4.25 ± 0.25 mV and 3.25 ± 0.5 mV respectively at 50 sec of irradiation.

**Figure 9:**
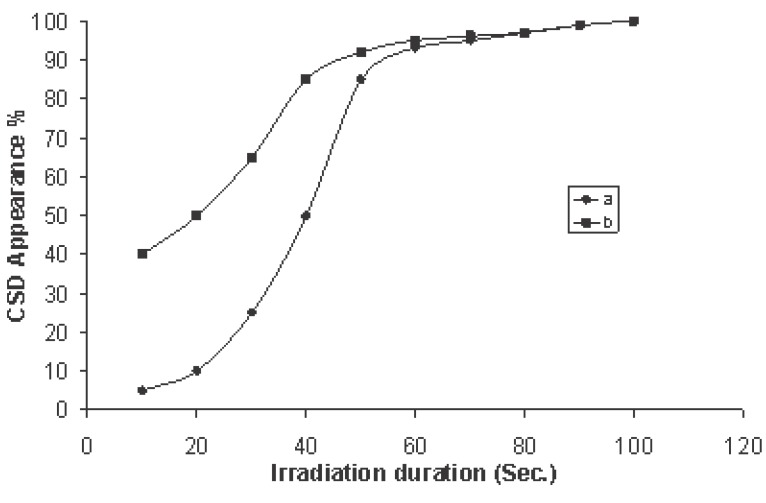
Appearance of CSD (%) with the irradiation duration of microwave exposure 10, 20, 30, 40 and 50 sec for (a) received signal to the mobile (b) transmitted signal from the mobile. (Each point on the curves represents a mean of five records).

Figure 9 shows the percentage of CSD appearance with increasing irradiation time. From observations during experimentation and the data analysis, the percent of appearance was 40% due to 10 sec irradiation time in case of transmitted signal exposure while it did not exceed 5% in case of received signal exposure. The percent of appearance showed an increase with increasing exposure time and reached 95% at 60 sec for both types of irradiation.

## DISCUSSIONS

Since Leão’s originals description in his initial papers (13, 14) and Garfstein (15), SPC represents an autoregenerative depolarization of brain cells with massive redistribution of jobs between intracellular and extracellular compartments. CSD has been the subject of several recent reviewers (3, 16, 17) and, for the importance of the subject, Garfstein return to the field in 2000.

In the present experiments, SD waves elicited by 2% KCl showed SPC which has values of amplitude, duration and propagation speed were in consistence with previous reviewers (1-3). SD can experimentally induced by a wide range of stimuli directly affecting the brain such as chemical agents, electrical current and mechanical stimuli. In the present work, microwave (935.2-960.2 MHz, 8.5 mW) from GSM mobile phone has been succeeded as an agent in evoking CSD in rats. The obtained results suggest that low level microwave from mobile phone can alter the membrane permeability of cortical cells which cause out flux of potassium and/or glutamate depolarizing adjacent cells continuing SD propagation. This interpretation agrees with the explanation given by Grafstein (15, 18) for the mechanism of CSD elicited by other agents. The release of SD transmitter at the site of stimulus application seems to be the factor common to the action of various chemical, electrical, mechanical and thermal stimuli. Since SD is initiated when the threshold amount of transmitter is liberated in critical volume of tissue and the efficiency of stimulus can be expressed by the mean amount of energy required to induce SD (1). It was found, in previous studies, that the threshold energies to produce SD were 20 Joule in normothermic rats by ultrasound agent, 0.01 Joule and 0.003 Joule by electrical and mechanical stimuli respectively (5). In present work the energy required to produce SD by microwave emitted from mobile phone was 0.418 Joule in the normothermic rats, which in comparison with the previous results speculate that microwave stimulus by mobile phone seems to resemble more the electrical stimulus in SD production mechanism.

The increase in amplitude of SPC with increasing the irradiation time noted in Fig. 5 and 6 is perhaps due to increase in the membrane permeability affected by the increase in microwave irradiation duration. Cleary *et al*. (19) reported that the flux of sodium and potassium ions across cell membrane can be affected by radio-frequency exposure, over a wide range of frequencies (27 MHz to 10 GHz). Many investigations in brain exposure to low level microwave and radiofrequency radiations showed alteration in the distinct aspects of the brain electrical activity accompanied by structural deformation in brain (20, 21). Microwave emitted by cellular phone at 900 MHz affected vascular permeability in mice brain (22).

SD waves illustrated in Fig. 5 and 6 showed SPCs (have relatively long duration) declined quiet early during the onset but was extremely slow to recover from the depression and this may indicate involvement of synapses or of interneuronal connections by the CSD (15, 18). The reason of the differences in amplitude and duration of SPC in case of transmitted signal exposure and received signal exposure could be attributed to the varying power density of the mobile device which is usually greater in case of transmitted signal than in case of received signal.
